# Post-viral alterations in periodontal health in individuals recovering from COVID-19

**DOI:** 10.3389/froh.2025.1654029

**Published:** 2025-08-15

**Authors:** Jelena Roganović, Milena Barać, Biljana Miličić, Milan Petrović, Stefan Sredojević, Ana Đinić Krasavčević, Nataša Nikolić-Jakoba

**Affiliations:** 1 Department of Pharmacology in Dentistry, Faculty of Dental Medicine, University of Belgrade, Belgrade, Serbia; 2 Department of Medical Statistics and Informatics, Faculty of Dental Medicine, University of Belgrade, Belgrade, Serbia; 3 Clinic for Maxillofacial Surgery, Faculty of Dental Medicine, University of Belgrade, Belgrade, Serbia; 4 Department of Periodontology, Faculty of Dental Medicine, University of Belgrade, Belgrade, Serbia

**Keywords:** periodontitis, post-COVID-19, epigenetics, saliva, methyl-transferase-like 3

## Abstract

This study explores post-viral immune modulation in periodontal health using COVID-19 convalescence as a model. We hypothesized that post-COVID-19 recovery induces epigenetic alterations, measurable through salivary methyl-transferase-like 3 (METTL3) expression and clinical-periodontal parameters. The present research comprises results from two studies: the clinical study, which included a total of 83 systemically healthy adults stratified into four groups according to periodontal status and COVID-19 history, and the laboratory study on human parotid gland samples (*n* = 10). Full-mouth periodontal status and unstimulated morning saliva were obtained. Glandular METTL3 and fat mass and obesity-associated factor as well as salivary METTL3 and cortisol were quantified using ELISA; psychological stress was assessed with the Perceived Stress Scale −10. Effect sizes were assessed using ANOVA and multivariable linear regression, and receiver operating characteristic (ROC) curves were generated for METTL3. Decreased levels of METTL3 in parotid tissue and in saliva of COVID-19 convalescents were found. Prior COVID-19 was significantly associated with METTL3 and plaque index (PI) as predictors, when adjusted for age, gender, periodontitis, and salivary cortisol. Strong associations between METTL3 and PI were found. Stress scores and cortisol did not differ between groups. Thus, downregulation of salivary METTL3 and concomitant plaque index reduction characterize the late convalescent phase of COVID-19. These epigenetic changes may reflect post-viral changes in parotid gland and periodontal health homeostasis and warrant longitudinal research confirmation.

## Introduction

In periodontitis, the host immune response is influenced by multiple factors, including microbial virulence, genetic predisposition, and environmental and systemic conditions (1). Among systemic influences, human viruses, due to their frequent presence in the oral cavity and their ability to disrupt immune homeostasis, are strong candidates for modulating periodontal health (2). The concomitant viral infection and post-viral immune disturbance represent two distinct phases of host–pathogen(s) interaction in periodontitis, each with different clinical and biological implications. During concomitant infection, viruses may exacerbate periodontal disease by directly damaging tissues, promoting bacterial dysbiosis, and intensifying local inflammation through immediate disruption of immune responses (2). However, investigations of post-viral effects are lacking, although they provide insight into sustained host adaptations that potentially affect periodontitis progression or treatment responsiveness. To address this gap, we investigated immune alterations during post-viral recovery using post-COVID-19 convalescence as a model, due to established SARS-CoV-2 oral tissue tropism, well-documented immune effects, and the availability of reliable diagnostic data (3). Bearing in mind that it has been reported that periodontitis-related epigenetic mechanisms are predicted to enhance viral entry pathways and modulate the host antiviral response related to COVID-19 (3), the most abundant epigenetic-like mechanism, N6-methyladenosine (m6A) RNA methylation, known to regulate gene expression during inflammation (4), becomes particularly relevant; thus, we selected methyl-transferase-like 3 (METTL3), a key m6A “writer,” for investigation, based on prior evidence indicating that its expression is specifically altered in response to SARS-CoV-2 infection. Namely, Zhang et al. used MeRIP-Seq and Nanopore DRS to demonstrate that only METTL3 and the m6A “eraser” fat mass and obesity-associated factor (FTO) were differentially expressed in SARS-CoV-2-infected cells, while other components of the m6A machinery remained unaffected (4). In the context of periodontitis, recent *in vitro* studies have shown that m6A-mediated RNA methylation patterns contribute to modulation of the immune microenvironment in periodontitis samples (5). Since tissue-based m6A signatures, although mechanistically informative, are clinically impractical, we aimed to explore the potential of a salivary assay as a more accessible approach to preliminarily examine possible links between post-viral immune modulation and periodontal inflammation. Specifically, we aimed to investigate the periodontal status of individuals recovering from COVID-19 and its association with salivary METTL3, considering that post-viral alterations in METTL3 expression may reflect broader immune and epithelial adaptations relevant to oral health after systemic viral infections.

## Material and methods

The present research comprises results from two studies conducted between September 2021 and November 2022. The clinical study included a total of 83 individuals who met the inclusion criteria and signed informed consent; all participants were recruited at the Department of Periodontology and Oral Medicine, Faculty of Dental Medicine. The laboratory study was conducted on human parotid gland specimens obtained from 10 additional individuals who underwent surgical treatment of the salivary glands and provided informed consent at the Department of Maxillofacial Surgery, Faculty of Dental Medicine. Due to the limited number of patients undergoing salivary gland surgery during the study period, and considering that these surgical cases involved benign salivary gland tumor, which could potentially influence salivary METTL3 expression, we decided to conduct two separate studies rather than combine patient data. The research was approved by the institutional Ethical Committee of the Faculty of Dental Medicine (number 36/10) and was registered on ClinicalTrials.gov (https://clinicaltrials.gov/ct2/home; ID number: NCT05205694).

### Clinical study

The cross-sectional study included systemically healthy individuals aged over 18 years, with or without a history of non-severe COVID-19. Those with prior infection in the late convalescent stage [1–6 months after symptom onset and a SARS-CoV-2-positive nasopharyngeal swab confirmed by real-time reverse transcription polymerase chain reaction (RT-PCR)]. Non-severe cases were defined as having no or mild pneumonia (less than 50% lung involvement on imaging within 24–48 h) and no dyspnea or hypoxia. Participants with periodontitis had stage II–IV disease (6), while periodontally healthy individuals showed no clinical signs of gingival inflammation, <10% bleeding sites, and probing depths ≤3mm. Exclusion criteria for all participants included smoking; systemic disease; use of immunosuppressants, antibiotics, or anti-inflammatory drugs in the past 6 months; current acute infection; pregnancy or lactation; fewer than 14 teeth present; and receipt of periodontal therapy within the last 6 months. Participants were classified into four groups: H group (periodontally healthy without a history of COVID-19), CH group ( periodontally healthy with a history of COVID-19), P group (periodontitis without a history of COVID-19), and CP group (periodontitis with a history of COVID-19). Periodontal status was determined by full-mouth examination using a PCPUNC 15 probe at six sites per tooth (mesiobuccal, mid-buccal, distobuccal, mesiolingual, mid-lingual, distolingual). All clinical measurements were conducted by an experienced periodontist. Intra-examiner calibration was performed twice—before and during the study—to ensure measurement consistency, achieving a Cohen's kappa value >0.75 for all clinical indices. Based on the observed effect size (Cohen's *f* = 0.45, derived from a pilot study with partial eta squared, *η*^2^ = 0.17) for differences in salivary METTL3 levels between groups, the required sample size was calculated using G*Power version 3.1. A total sample of 60 participants (15 per group) was required to achieve a statistical power of 0.80 and a significance level of α = 0.05 using one-way ANOVA for independent groups.

### Salivary sampling

From recruited individuals, a fasting 8 a.m. sample of unstimulated saliva was collected using the “spitting method.” Cortisol (Cortisol Parameter Assay Kit, Bio-Techne Ltd, Abingdon, UK) and METLL3 (Human METTL3 ELISA Kit, Assay Genie, Dublin, Ireland) were measured in saliva by ELISA according to the manufacturer’s instructions.

### Questionnaires

Demographic data and data on the presence and duration of COVID-19 symptoms were gathered by questionnaire. The level of psychological stress was estimated by a 10-item Perceived Stress Scale, translated into Serbian and validated in Serbian samples (7).

### Laboratory study

Fragments of human parotid glands were obtained from another 10 patients, with and without prior COVID-19, aged >50 years, who underwent parotidectomy due to benign parotid tumor, with informed consent. Surgically removed parotid tissue without histopathological signs of alterations was used in the study, and samples were transported in medium and then stored. Tissue samples were homogenized, and the resulting supernatants were used in ELISA assays to measure METTL3 and human FTO using ELISA kits (ELK Biotechnology, Denver, CO, USA).

### Statistics

The data were collected and managed using SPSS Version 28.0 (IBM Corp., Armonk, NY, USA). The results were presented as mean ± SD. The chi-square test was applied for testing categorical variables. One-way ANOVA or non-parametric test was used to assess significant differences among the groups and a Mann–Whitney test was used to compare the two groups/parameters. *p* < 0.05 was considered statistically significant. Multivariate regression analyses were used to investigate whether the participants' characteristics, periodontal and salivary parameters, or perceived stress were significantly associated with or predicted COVID-19. To evaluate the accuracy of METTL3 in differentiating COVID-19 convalescents, the receiver operating characteristic (ROC) curves were constructed by calculating the area under the curve (AUC) and identifying cutoff points to estimate the highest sensitivity and specificity.

## Results

### METTL3 and FTO in parotid gland tissue of patients with or without prior COVID-19

**Figure 1:**
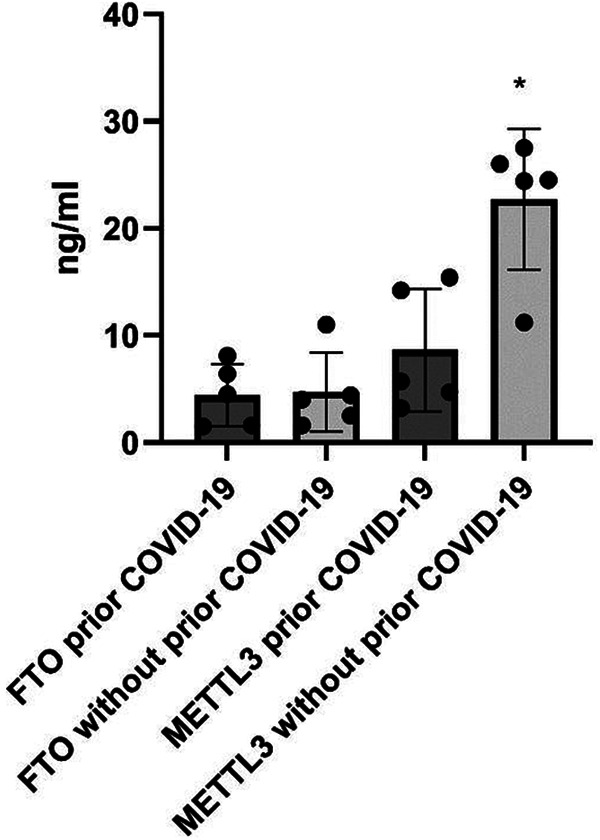
Expression levels of METTL3 and demethylase FTO in parotid glands from patients with/without prior COVID-19. *p < 0.05, with vs. without prior COVID-19, Mann–Whitney U-test.

In the parotid glands of patients with a prior confirmed COVID-19 diagnosis, only METTL3 expression levels were significantly decreased (8.6 ± 5.7 vs. 22.7 ± 6.5 ng/mL; *p* = 0.03), whereas FTO levels showed no significant difference (4.4 ± 2.9 vs. 4.7 ± 3.7 ng/mL) (Figure 1).

### Periodontal parameters in patients with a history of COVID-19

Measurements of periodontal parameters revealed a reduction in the periodontitis group with prior COVID-19 infection compared to those without previous viral infection (Table 1), along with a significant decrease in salivary METTL3 expression (*p* = 0.0003) (Table 1). Multivariable linear regression analysis identified prior COVID-19 and plaque index (PI) as significant predictors of METTLE3 levels (*p* = 0.001 and 0.04, respectively), after adjusting for age, gender, periodontitis status, and salivary cortisol (Table 2). In the CH group, a significant correlation was observed between METTL3 and PI (Spearman *r* = 0.39, *p* = 0.044). In a separate multivariable linear regression model assessing predictors of salivary METTL3 expression, prior COVID-19 remained the only statistically significant variable (*p* = 0.0008), when adjusted for age, gender, periodontitis, and other clinical parameters (Table 2). Notably, perceived stress scores and salivary cortisol levels did not differ significantly between the groups (Table 1).

**Table 1:** Post-viral periodontal health and salivary markers: periodontal parameters and salivary markers in COVID-19 convalescents.

Characteristics	H group (*N* = 23)	CH group (*N* = 22)	P group (*N* = 22)	CP group (*N* = 16)
Age (mean ± SD)	26.3 ± 4.0	25.4 ± 3.8	45.9 ± 11.5	51.9 ± 10.2
PPD (mean ± SD)	2.1 ± 0.3	2.3 ± 0.3	3.4 ± 0.8	2.8 ± 0.3**
CAL (mean ± SD)	0.0	0.0	3.5 ± 1.2	2.7 ± 0.8**
BOP (%)	7.5 ± 1.8	5.5 ± 2.9*	57.3 ± 23.6	41.6 ± 24.6*
PI (%)	38.9 ± 16.1	27.3 ± 23.4	73.4 ± 26.0	44.7 ± 33.8*
METTL3 (ng/mL)	11.1 ± 6.6	3.3 ± 1.8**	7.3 ± 5.1	6.6 ± 4.6
Cortisol (ng/mL)	44.1 ± 22.1	45.2 ± 22.1	44.8 ± 15.3	39.2 ± 17.2
Perceived stress (score)	16.4 ± 6.3	16.2 ± 4.5	15.0 ± 6.4	14.4 ± 4.6

H group, periodontally healthy participants without history of COVID-19; CH group, participants without periodontitis and with history of COVID-19; P group, participants with diagnosed periodontitis but no history of COVID-19; CP group, participants with diagnosed periodontitis and history of COVID-19. BOP, bleeding on probing; CAL, clinical attachment level; PI, plaque index; PPD, pocket probing depth.

* *p* < 0.05.

** *p* < 0.01 patients with vs. without history of COVID-19 (ANOVA, Kruskal–Wallis).

**Table 2:** Multiple linear regression analyses of the association between salivary METTL3 and prior COVID-19, adjusted for age, gender, and periodontitis.

Dependent variable	Independent variable	Estimate	Standard error	95% CI (asymptotic)	*p*-value
METTL3	Gender	−2.44	1.40	−5.24 to 0.36	0.09
	Age	−1.17	1.93	−5.03 to 2.69	0.55
	Prior COVID-19	4.51	1.29	1.94 to 7.08	0.0008
	Periodontitis	2.87	2.06	−1.24 to 6,97	0.17
	BOP	0.07	0.04	−0.01 to 0.15	0.08
	PI	−0.03	0.03	−0.09 to 0.03	0.29
Prior COVID−19	Gender	0.09	0.13	−0.17 to 0.35	0.50
	Age	0.25	0.17	−0.09 to 0.60	0.15
	Periodontitis	0.13	0.19	−0.26 to 0.52	0.50
	BOP	−0.001	0.004	−0.009 to 0.006	0.70
	PI	0.005	0.002	0.0003 to 0.01	0.04
	Cortisol	0.001	0.003	−0.005 to 0.006	0.73
	METTL3	0.04	0.01	0.01 to 0.06	0.001

BOP, bleeding on probing; PI, plaque index; PPD, pocket probing depth.

### Salivary METTL3 as a diagnostic marker of prior COVID-19

**Figure 2:**
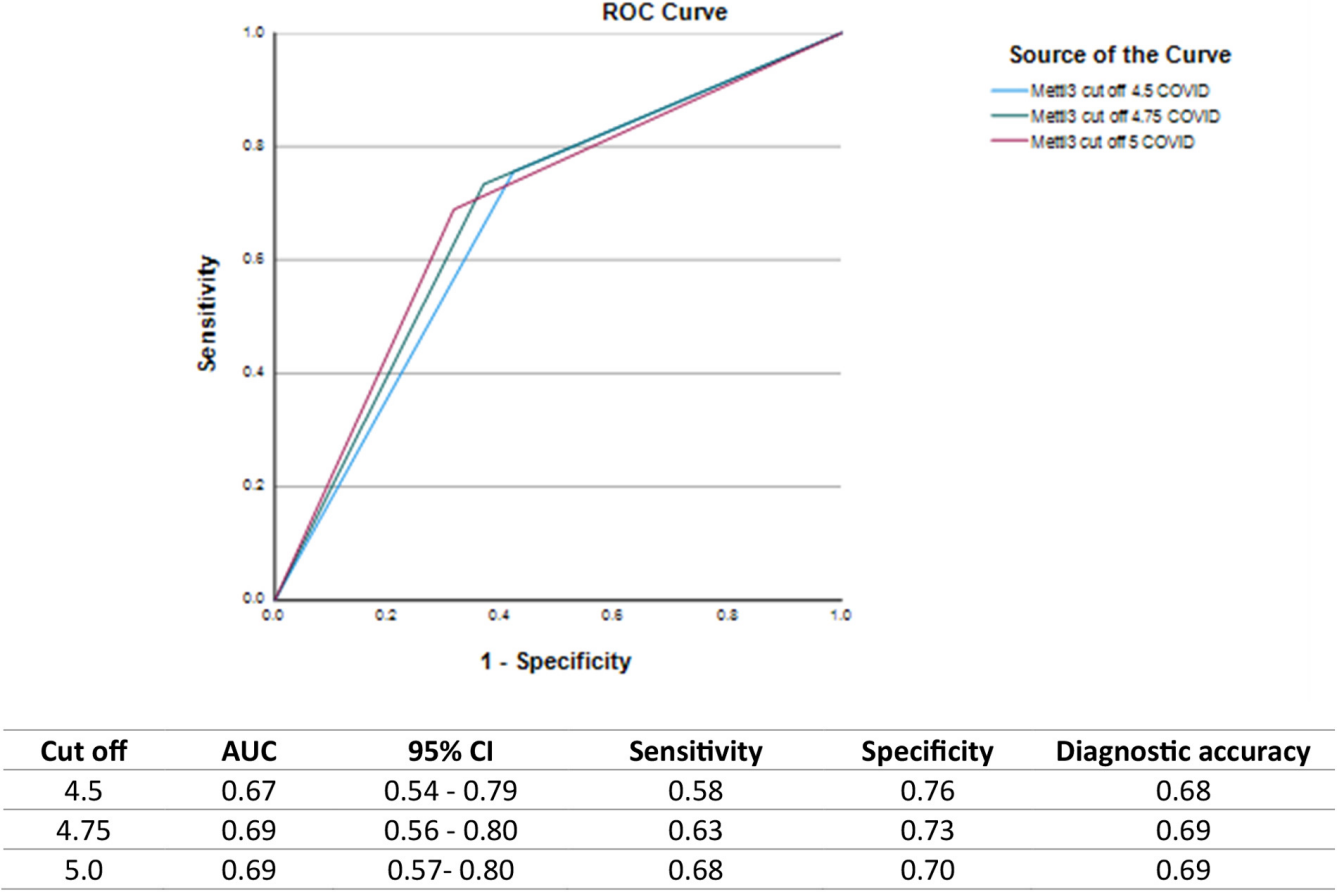
ROC curves and diagnostic performance of salivary METTL3 levels for identifying individuals with prior COVID-19 infection at different cutoff points. ROC curves illustrate the diagnostic ability of salivary METTL3 at cutoff values of 4.5, 4.75, and 5.0 ng/mL along with corresponding values for AUC, 95% confidence intervals (CI), sensitivity, specificity, and diagnostic accuracy.

Salivary METTL3 is a biomarker with the ability to identify COVID-19 survivors, with a moderate accuracy of 0.69 and a cutoff value of 4.75 (Figure 2).

## Discussion

In periodontitis, where disease progression depends on a delicate balance between microbial challenge and host response, even subtle changes in immune regulation can affect progression or resolution of periodontal inflammation. After viral clearance, the immune system may not return to its pre-infection baseline, but instead may undergo persistent immune modulation, including alterations in regulatory pathway signaling (2, 3). Consistent with this possibility, the present results show that individuals with prior viral infection exhibit a distinct oral signature: they show both an epigenetic-immunomodulatory change (decreased METTL3) and lower plaque accumulation. Namely, these two factors distinguished patients with prior COVID-19 from those without, even after adjusting for age, gender, and periodontal status. In patients with prior infection, they also showed a strong mutual association. The measurable changes in METTL3 and PI during the recovery phase support the idea of lasting post-viral immune adaptation rather than an acute-phase inflammatory response. Although salivary METTL3 may originate from several sources, including salivary gland epithelial cells, oral mucosal epithelium, and immune cells, the observed reduction in METTL3 expression in the parotid gland, along with the predominant contribution of glandular secretion to resting saliva, suggests that the decreased salivary METTL3 levels primarily reflect post-viral downregulation within the salivary glands.

Given that METTL3 is induced by and interacts with SARS-CoV-2 viral proteins (4), its reduced expression may reflect a consequence of viral clearance. This clearance likely triggered a shift toward immune resolution in the oral environment, characterized by the restoration of epithelial integrity and the rebalancing of cytokine and antimicrobial peptide profile (8). Such changes may have suppressed bacterial overgrowth and biofilm formation (9), resulting in lower clinical plaque indices, even in the absence of improved oral hygiene practices. The observed decrease in clinical plaque is unexpected, as one might assume that compromised health or disrupted daily routines during or after illness would lead to poorer oral hygiene (10). One possible explanation is that individuals recovering from COVID-19 became more attentive to personal hygiene, including oral hygiene, due to heightened health awareness during the pandemic. However, results are conflicting so far (11, 12). In addition, altered dietary patterns (e.g., softer or less cariogenic foods during convalescence) and reduced exposure to environmental factors may have indirectly contributed to lower plaque accumulation. Currently, COVID-19 convalescents showed markedly decreased salivary METTL3 levels, which is largely explained by their prior viral infection, and not by age, sex, or baseline periodontal status. Thus, viral exposure appears to induce epigenetic changes that alter the host-microbial balance in the oral environment, influencing periodontal parameters—especially plaque accumulation. The positive correlation observed between PI and METTL3 in participants with prior COVID-19 but without periodontitis suggests that SARS-CoV-2 convalescence modifies the baseline activity of the m6A machinery, enhancing its responsiveness to minimal microbial stimuli: even minimal variations in biofilm raise METTL3 expression to suppress inflammatory signs such as bleeding on probing (BOP). Thus, it seems that METTL3 may function as a rapid epigenetic sensor of microbial challenge in a host whose immune set-point has been recalibrated after viral infection. Although reduced plaque accumulation in COVID-19 convalescents may reflect these post-viral immune modifications, alternative explanations—such as changes in salivary composition or shifts in the oral microbiome—cannot be excluded and likely contribute to the observed pattern.

Although stress is known to contribute to immune dysregulation after viral infections, and glucocorticoid signaling is influenced by m6A/METTL3 expression (13), our participants did not exhibit significant changes in self-perceived stress scores or salivary cortisol levels. Moreover, these stress-related measures were not associated with salivary METTL3 expression.

The m6A modification, regulated by the methyl-transferase complex containing METTL3, plays a critical role in immune responses to RNA viruses such as coronaviruses, rhinoviruses, and influenza (14)—infections commonly encountered by dental patients. These viral infections may transiently disrupt the local microbiome and mucosal immunity, leaving a post-viral inflammatory imprint on the oral environment (5). To date, few studies have connected respiratory virus convalescence with oral health via an epigenetic link, thus demonstrating this provides dentists with a rationale to monitor convalescent patients more closely and highlighting the potential for host-modulating therapies targeting METTL3. However, further longitudinal research is needed to confirm these observations.

## Data Availability

Original datasets are available in a publicly accessible repository: The original contributions presented in the study are publicly available. This data can be found here: https://doi.org/10.6084/m9.figshare.29403680.

## References

[1] Chen TP Yu HC Lin WY Chang YC. The role of microbiome in the pathogenesis of oral-gut-liver axis between periodontitis and nonalcoholic fatty liver disease. J Dent Sci. (2023) 18:972–5. 10.1016/j.jds.2023.03.012 37404621 PMC10316499

[2] Teles F Collman RG Mominkhan D Wang Y. Viruses, periodontitis, and comorbidities. Periodontol 2000. (2022) 89:190–206. 10.1111/prd.12435 35244970

[3] Roganović JR. microRNA-146a and -155, upregulated by periodontitis and type 2 diabetes in oral fluids, are predicted to regulate SARS-CoV-2 oral receptor genes. J Periodontol. (2021) 92:35–43. 10.1002/JPER.20-0623 33336412

[4] Zhang X Hao H Ma L Zhang Y Hu X Chen Z Methyltransferase-like 3 modulates severe acute respiratory syndrome coronavirus-2 RNA N6-methyladenosine modification and replication. mBio (2021) 12:e01067-21. 10.1128/mbio.01067-21 34225491 PMC8437041

[5] Zhang X Zhang S Yan X Shan Y Liu L Zhou J M6a regulator-mediated RNA methylation modification patterns are involved in immune microenvironment regulation of periodontitis. J Cell Mol Med. (2021) 25:3634–45. 10.1111/jcmm.16469 33724691 PMC8034465

[6] Tonetti MS Greenwell H Kornman KS. Staging and grading of periodontitis: framework and proposal of a new classification and case definition. J Periodontol. (2018) 89(1):S159–72. 10.1002/JPER.18-0006 29926952

[7] Jovanović V Gavrilov-Jerković V. More than a (negative) feeling: validity of the perceived stress scale in Serbian clinical and non-clinical samples. Psihologija. (2015) 48:5–18. 10.2298/PSI1501005J

[8] Johnstone KF Herzberg MC. Antimicrobial peptides: defending the mucosal epithelial barrier. Front Oral Health. (2022) 3:958480. 10.3389/froh.2022.958480 35979535 PMC9376388

[9] Abdulkareem AA Al-Taweel FB Al-Sharqi AJB Gul SS Sha A Chapple ILC. Current concepts in the pathogenesis of periodontitis: from symbiosis to dysbiosis. J Oral Microbiol. (2023) 15::2197779. 10.1080/20002297.2023.2197779 37025387 PMC10071981

[10] Dickson-Swift V Kangutkar T Knevel R Down S. The impact of COVID-19 on individual oral health: a scoping review. BMC Oral Health. (2022) 22(1):422. 10.1186/s12903-022-02463-0 36138456 PMC9502893

[11] Guerreiro E Cachinho R Dionísio T Nobre M Júdice A Simões C Oral health and dietary habits before and after COVID-19 restrictions in a Portuguese adult population: an observational study. Life. (2025) 15(5):746. 10.3390/life15050746 40430174 PMC12113164

[12] Hatipoğlu Palaz Z Aktaş N Atabek D. The impact of the COVID-19 pandemic on the oral hygiene Status of children with high caries risk and their parents. COVID. (2024) 4(12):1897–907. 10.3390/covid4120133

[13] Zhao Y Jiang Y Feng Y Zhao R. RNA m6A-mediated post-transcriptional repression of glucocorticoid receptor in LPS-activated Kupffer cells on broilers. Poult Sci. (2025) 104:104393. 10.1016/j.psj.2024.104393 39571201 PMC11617446

[14] Liu X Chen W Li K Sheng J. RNA N6-methyladenosine methylation in influenza A virus infection. Front Microbiol. (2024) 15:1401997. 10.3389/fmicb.2024.1401997 38957616 PMC11217485

